# 976. Population Pharmacokinetic/Pharmacodynamic (PK/PD) Target Attainment Analysis of Intravenous Fosfomycin for Treatment of Multidrug-Resistant Gram-Negative Bacterial Infections

**DOI:** 10.1093/ofid/ofad500.031

**Published:** 2023-11-27

**Authors:** Walaiporn Wangchinda, jason M Pogue, Visanu Thamlikitkul, Pannee Leelawattanachai, Pornpan Koomanachai, Manjunath (Amit) Pai

**Affiliations:** University of Michigan College of Pharmacy, Ann Arbor, MI; University of Michigan, College of Pharmacy, Ann Arbor, Michigan; Faculty of Medicine Siriraj Hospital, Mahidol University, Bangkok, Krung Thep, Thailand; Navamindradhiraj University, Bangkok, Krung Thep, Thailand; Faculty of Medicine Siriraj Hospital, Mahidol University, Bangkok, Krung Thep, Thailand; Clinical Pharmacy, University of Michigan

## Abstract

**Background:**

Intravenous (IV) fosfomycin is increasingly used globally for treatment of infections due to multidrug-resistant Gram-negative bacteria (GNB). This study aimed to determine the optimal dosage of IV fosfomycin for patients with varying degrees of kidney function.

**Methods:**

Eligible patients were adults ≥ 18 years who were treated with IV fosfomycin 2-4 g every 6-8 hours. Five serial blood samples were collected after the 4^th^ dose of fosfomycin and concentrations were measured and modeled by population PK analysis. Monte Carlo simulations were conducted to evaluate the probability of target attainment (PTA) of different dosages using species-specific 1-log_10_ kill targets (AUC_24h_/MIC of 98.9, 21.5, and 28.2 for *E. coli*, *K. pneumoniae*, and *P. aeruginosa*, respectively). The kidney function adjusted dosing regimens for *E. coli* are proposed by using the lowest dose that can achieve ≥ 90% PTA at an MIC of 32 (EUCAST breakpoint) and 64 µg/mL. The highest MIC values at which selected doses provide ≥ 90% PTA for *K. pneumoniae* and *P. aeruginosa* were also analyzed.

**Results:**

A total of 138 plasma samples from 28 patients were included. The model was best described by a 1-compartment model with linear elimination. Significant covariates in final model were creatinine clearance and male sex on clearance (CL), and body weight on volume of distribution (*V*). The parameter estimates were CL of 0.65 ± 0.1 L/h, and *V* of 12.59 ± 0.8 L. The PTA of simulated dosages for *E. coli* are shown in Figure 1. For MIC ≤ 32 mcg/mL, a dosage of 12 g/day achieved ≥ 90% PTA for CL_CR_ 90-120 ml/min. For MIC ≤ 64 µg/mL, a dosage of 24 g/day was needed. The proposed kidney function adjusted dosing regimens for *E. coli* are shown in Table 1. The highest MIC values of *K. pneumoniae* and *P. aeruginosa* where these doses provide ≥ 90% PTA were 128 and 256 µg/mL, respectively.

**Figure:**
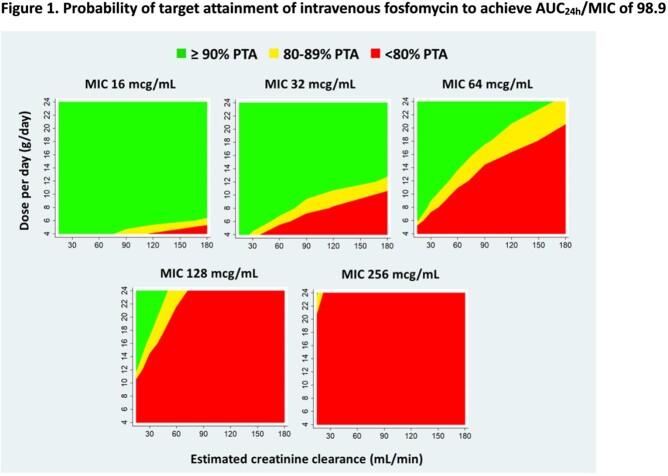

**Figure:**
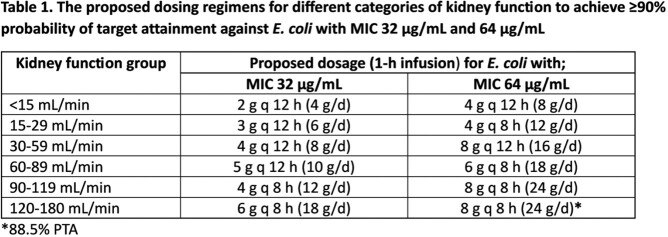

**Conclusion:**

A dosage of 12 g/day of fosfomycin is required for *E. coli* at EUCAST susceptible breakpoint in patients with normal renal clearance and dose adjustments are needed for patients with renal insufficiency.

**Disclosures:**

**jason M. Pogue, PharmD**, AbbVie: Advisor/Consultant|Entasis: Advisor/Consultant|Ferring: Advisor/Consultant|GSK: Advisor/Consultant|Merck: Advisor/Consultant|Merck: Grant/Research Support|Qpex: Advisor/Consultant|Shionogi: Advisor/Consultant

